# Y-Chromosomal Variation in Uyghurs from Kazakhstan Reveals High Paternal Diversity and Close Affinity with Xinjiang Uyghurs

**DOI:** 10.3390/ijms27198641

**Published:** 2026-09-27

**Authors:** Maxat Zhabagin, Alizhan Bukayev, Ayagoz Mailenova, Aigul Zhunussova, Zhanargul Dyussenova, Almas Toxanbayev, Zhaxylyk Sabitov

**Affiliations:** 1 National Center for Biotechnology, Astana 010000, Kazakhstan; 2 DNK Shejire LLP, Astana 010000, Kazakhstan; 3 Research Institute for Jochi Ulus Studies, Astana 010000, Kazakhstan; 4 Kazak Historical Society, Astana 010000, Kazakhstan

**Keywords:** Y-chromosome, Y-STR, haplotype diversity, median-joining network, Uyghurs, Central Asia

## Abstract

Y-chromosomal reference data from Uyghur communities outside China remain limited, although these populations are central to understanding paternal genetic structure in Central Asia. We analysed 596 reportedly unrelated Uyghur males from four regions of southeastern Kazakhstan using 27 Y-STR loci and 20 core Y-SNP markers, and compared the resulting haplotypes with Uyghur and neighbouring Central Asian populations from the Y-Chromosome Haplotype Reference Database. The dataset yielded 515 distinct haplotypes, high overall haplotype diversity (0.9993), and a low observed haplotype match probability (0.0023), providing a robust baseline for forensic applications. The paternal pool was highly heterogeneous, with major contributions from R1a (19.97%), J2 (17.45%), C2 (12.25%) and O2 (10.74%) lineages, alongside additional minor western, eastern and northern Eurasian components. Median-joining networks displayed complex, multi-branched topologies, while an exploratory comparison with a separate, previously published NGS dataset (*N* = 19) confirmed that these broad haplogroups contain multiple phylogenetically distinct subclades. Pairwise RST and nonmetric multidimensional scaling (NMDS) analyses detected no statistically significant differentiation among Kazakhstani Uyghur regional groups and Xinjiang Uyghur populations after correction for multiple testing, whereas several Kazakh and Kyrgyz groups were more clearly differentiated. These results establish a highly discriminatory regional forensic reference dataset and position Kazakhstani Uyghurs within a cohesive Central Asian paternal continuum shaped by diverse multiple male-mediated inputs.

## 1. Introduction

The non-recombining region of the human Y chromosome (NRY) provides information on paternal genetic variation because it is inherited largely as a single linked unit [1]. Slowly mutating Y-chromosomal single-nucleotide polymorphisms (Y-SNPs) define phylogenetic branches, whereas more rapidly mutating Y-chromosomal short tandem repeats (Y-STRs) provide finer resolution among paternal lineages and support the development of population reference datasets and forensic applications [2]. Y-STR loci are not statistically independent and paternal relatives may share the same haplotype; consequently, Y-STR match statistics must be interpreted as lineage-based evidence rather than as autosomal random match probabilities [3]. The Y-Chromosome Haplotype Reference Database (YHRD) supports quality-controlled haplotype frequency searches and population comparisons [4], but high-resolution reference datasets for Central Asian populations remain comparatively limited.

Central Asia has long been a major region of trans-Eurasian population movement. Early Y-chromosomal studies highlighted the region as a major reservoir of genetic diversity shaped by population movements across Eurasia [5]. More recent genome-wide analyses have shown that the genetic landscape of Central Asia was shaped by repeated interactions among populations with steppe, Siberian, East Asian, and Iranian-related populations [6]. Ancient DNA studies have further documented complex patterns of population turnover and admixture across the region [7]. Xinjiang, in particular, is a key contact zone between eastern and western Eurasia, with a long history of gene flow and cultural exchange associated with prehistoric population movements and Silk Road networks.

Within broader region, the Uyghurs are a well-studied example of an admixed population. Genome-wide analyses have identified substantial ancestry contributions from both eastern and western Eurasian sources [8]. Subsequent studies refined this model by identifying additional Siberian and South Asian ancestry components [9]. Further analyses have linked this admixture to multiple episodes of historical contact across Eurasia [10]. Admixture patterns also vary geographically within Xinjiang, with regional differentiation along southwest–northeast gradients associated with geography-mediated gene flow and repeated episodes of population contact [11].

Y-chromosomal data are broadly consistent with these findings. Analyses based on Y-STR panels have shown that paternal genetic variation in Uyghurs is characterized by high haplotype diversity and genetic affinities to East, western Eurasian and Central Asian populations [12]. Additional studies support diverse paternal ancestry rather than a single predominant founder lineage [13].

Despite these advances, published research has focused primarily on Uyghur populations in Xinjiang. Uyghur communities in Kazakhstan have received comparatively little attention in high-resolution studies of paternal genetics. This gap is particularly relevant because Kazakhstan hosts one of the largest Uyghur populations outside China, with 290,337 individuals recorded in the 2021 national census [14]. These communities developed through multiple waves of migration from Xinjiang. The first major wave involved resettlement in the late 19th century following the Ili crisis [15]. Further population movements in the early 20th century were driven by sociopolitical instability and the suppression of national movements in Xinjiang [16]. Another major influx occurred in the mid-20th century, particularly during 1950s and 1960s, amid changes in Sino-Soviet relations [17]. These successive migration waves contributed to the demographic diversity of Uyghur communities in Kazakhstan, as described in historical and ethnographic studies.

Recent studies have expanded Y-chromosomal reference datasets for Kazakh populations using panels of 27 Y-STR markers, providing a comparative framework for regional analyses [18]. Additional work has improved phylogenetic resolution and further characterized the population structure of Central Asian Y-chromosomal variation [19]. These datasets provide an opportunity to assess whether Uyghurs in Kazakhstan exhibit distinctive patterns of paternal genetic variation or share patterns observed in other Central Asian populations.

This study investigates the paternal genetic diversity and structure of Uyghurs from Kazakhstan using a high-resolution panel of 27 Y-STR markers. By combining haplotype analysis with comparisons among populations, we aim to characterize paternal lineage diversity, examine haplotype clustering, and assess genetic relationships between Kazakhstani Uyghurs and other Central Asian populations. These data provide a reference for forensic applications and contribute to the study of male-mediated population history in the region.

## 2. Results and Discussion

The newly genotyped sample comprised 596 Uyghur males from four sampling locations in southeastern Kazakhstan (Figure 1): Almaty city (*N* = 179), Enbekshikazakh District (*N* = 203), Panfilov District (*N* = 142), and Uyghur District (*N* = 72). All 596 individuals were genotyped at 27 Y-STR markers and 20 Y-SNP markers used to assign major haplogroups (Supplementary Table S1). A separate dataset comprised previously published next-generation sequencing (NGS) data from 19 individuals [19].

### 2.1. Analysis of Haplotype and Allele Diversity

The complete 27-marker Y-STR haplotypes are provided in Supplementary Table S1. Table 1 summarizes the number of distinct haplotypes, the singleton proportion, discrimination capacity (DC), observed haplotype match probability (HMP), and unbiased haplotype diversity (HD).

Among the 596 newly genotyped individuals, 515 distinct 27-marker haplotypes were observed, of which 454 occurred once (singleton proportion = 0.7617). In the pooled sample, DC was 0.8641, HMP was 0.00235, and HD was 0.99932 (Table 1; Supplementary Tables S1 and S2).

Haplotype diversity was high across the four sampling locations, although the other parameters varied. The numbers of distinct haplotypes were 170 in Almaty, 187 in Enbekshikazakh District, 132 in Panfilov District, and 62 in Uyghur District. The corresponding singleton proportions were 0.9050, 0.8522, 0.8803, and 0.7639, respectively. HD ranged from 0.99452 in Uyghur District to 0.99937 in Almaty. Observed HMP ranged from 0.00585 to 0.01929 across the four samples and was 0.00235 in the pooled sample. DC ranged from 0.8611 to 0.9497.

Allele frequencies and gene diversity (GD) values for the 23 single-copy loci are reported in Supplementary Table S3. Frequencies of unordered allele pairs at the multicopy loci DYS385 and DYF387S1 are reported in Supplementary Table S4. Figure 2 shows the allele frequency distributions at the single-copy loci.

A total of 197 distinct allele designations were observed across the single-copy loci. The number of alleles per locus ranged from five at DYS391, DYS393, DYS437, and DYS460 to 16 at DYS458.

GD varied markedly among loci (Supplementary Table S3), ranging from 0.496 at DYS391 to 0.870 at DYS449. High GD values were also observed at DYS518 (0.865), DYS627 (0.861), and DYS481 (0.844), whereas lower values were observed at DYS437 (0.550) and DYS533 (0.632).

At the multicopy loci, 65 distinct unordered allele pairs were identified at DYS385 and 42 at DYF387S1. Both loci showed high gene diversity, with GD values of 0.956 and 0.942, respectively. The most frequent allele pairs were 11, 14 at DYS385 and 37, 38 at DYF387S1, each with a frequency of 0.149.

Confirmed atypical allele calls are listed in Supplementary Table S5. Microvariant alleles were most frequently observed at DYS458 (*N* = 29). Reproducible amplification failure, suggestive of null alleles, was observed primarily at DYS448 (*N* = 13) and less frequently at DYS456 (*N* = 2) and Y-GATA-H4 (*N* = 2). Additional microvariants were observed at DYS518 (*N* = 9), while copy number variants and allele duplications were observed at DYS19 (*N* = 7). Other atypical observations included single occurrences of the allele combinations 14, 15 at DYS389I and 32, 33 at DYS389II, the microvariant 19.2 at DYS627, and the allele combination 38, 41.2 at DYF387S1. Two additional rare alleles, 18.4 and 20.2, were observed at DYS448 (n = 2).

### 2.2. Median-Joining Network Analysis

To explore relationships among Y-STR haplotypes, we constructed a median-joining network using 15 loci: DYS19, DYS389I, DYS389II, DYS390, DYS391, DYS392, DYS393, DYS437, DYS438, DYS439, DYS448, DYS456, DYS458, DYS635, and Y-GATA-H4. DYS385a/b was excluded because its multicopy nature complicates allele assignment and may introduce ambiguity into network reconstruction. Additional loci—DYS449, DYS481, DYS518, DYS570, DYS576, DYS627, and DYF387S1a/b—were also excluded to reduce reticulation and simplify the network.

The resulting network (Figure 3) shows a complex, moderately reticulated structure, consistent with the high haplotype diversity observed in the sample. It comprises multiple interconnected clusters rather than a single predominant cluster, providing a descriptive overview of paternal haplotype variation.

Several major clusters correspond to the predominant Y-chromosomal haplogroups (Supplementary Table S1, Figure 1). The most prominent cluster is associated with R1a (*N* = 119; 19.97%) and forms a relatively compact group with short mutational branches. J2 (*N* = 104; 17.45%) shows a more dispersed configuration with multiple sub-branches. Additional clusters correspond to O2 (*N* = 64; 10.74%) and C2 lineages (*N* = 73; 12.25%). R1b (*N* = 47; 7.89%), J1 (*N* = 37; 6.21%), G (*N* = 31; 5.20%) and Q (*N* = 31; 5.20%), are represented by smaller clusters or peripheral nodes. Other haplogroups, including N1, E, D, L, and T, occurred at low frequencies in the full sample and contributed to its overall haplogroup diversity.

For comparison, previously published data on Xinjiang Uyghurs showed a broadly similar haplogroup composition, although relative frequencies differed (Figure 1) [10]. R1a was also the most frequent haplogroup, accounting for 30.1% of the sample, followed by O2 (14.0%), J2 (10.8%), R1b and F (7.5% each), C2 (6.5%), and R2 (5.4%). Lower frequencies were reported for E (4.3%), G and N1 (3.2% each), D and Q (2.2% each), and C1, L, and O1 (1.1% each); H, I, J1, and T were not detected. Both datasets therefore show a heterogeneous haplogroup composition, with lineages commonly associated with western Eurasia, including R1a, J2, and R1b, as well as lineages commonly associated with eastern Eurasia, including O2 and C2.

To examine within-haplogroup variation in greater detail, we constructed separate median-joining networks for R1a and J2, the two most frequent haplogroups (Figure 4). The R1a network (Figure 4A) shows a broad, highly branched topology with several internal nodes and multiple low-frequency terminal haplotypes, indicating substantial haplotype variation within this haplogroup. Haplotypes from Almaty city and Enbekshikazakh, Panfilov, and Uyghur districts are intermingled throughout the network, with no visually distinct geographic clusters.

The J2 network (Figure 4B) shows reticulated structure with several subclusters connected by longer mutational branches. Relative to the R1a network, it appears more dispersed, with more pronounced subclustering.

Both networks show substantial within-haplogroup haplotype variation, although their configurations differ. The geographic intermingling of haplotypes is compatible with shared paternal ancestry among the sampled communities, but network topology alone does not establish the extent or timing of gene flows.

### 2.3. Exploratory Analysis of Previously Published Y-Chromosome Sequencing Data

To place the patterns observed in the primary sample in a broader phylogenetic context, we examined previously published NGS-based Y-SNP data from Uyghur males in Kazakhstan [19]. The published haplogroup assignments were re-evaluated using the Y-chromosome phylogeny available in FamilyTreeDNA Discover (https://discover.familytreedna.com/ (accessed on 1 April 2026)) as an auxiliary comparative resource to refine the phylogenetic placement of individual lineages (Supplementary Table S6). Because these 19 individuals were not sampled to represent the wider population and the public phylogenetic resources are continually updated, this analysis is exploratory and hypothesis-generating. The NGS data were analyzed separately from the primary sample of 596 individuals and were not used to estimate population haplogroup frequencies.

The separate NGS dataset comprised 19 Kazakhstani Uyghur males assigned to eight broad haplogroup categories: C2 and R1a were each represented by four individuals, O by three. G2, J and Q1 by two each, and N1 and R1b by one each. At finer phylogenetic resolution, the individuals were assigned to distinct subclades, revealing diversity within several broad haplogroups. These observations illustrate how broad haplogroup categories can encompass multiple phylogenetically distinct paternal lineages.

The four R1a lineages all belonged to R1a-Z93. In the reference phylogeny, R1a-BY65786 is positioned near lineages reported from West Asian populations in Iraq, Qatar, and Kuwait, indicating a comparative phylogenetic affinity with lineages sampled in that region. R1a-Y186589 clusters with a Dungan individual from Kazakhstan, while its sister branch, R1a-FTJ41090, includes several ancient individuals reported in [20] and included in the Allen Ancient DNA Resource (AADR) [21]. The other two lineages, R1a-FTJ191 and R1a-Y148183, fall within R1a-S23592. R1a-Y148183 belongs to the broader R1a-YP1564 clade, which includes reported Kyrgyz Y-chromosomal lineages. R1a-FTJ191 clusters with the ancient individual I19084 from the Brusyany-2 burial ground in the Samara region of Russia, dated to the early medieval period and attributed to the Novinki archaeological context [22]. Although the Novinki cultural horizon is often discussed in relation to early medieval steppe populations and the Khazar Khaganate, this phylogenetic affinity indicates shared ancestry rather than a direct migration event. The observed R1a variation therefore comprises several distinct lineages within R1a-Z93.

One individual belonged to R1b-FTJ40717, a subclade of R1b-M73. This lineage clusters with a Nogai individual in the reference phylogeny, adding a further Inner Eurasian phylogenetic affinity to the NGS dataset. Its occurrence documents an additional branch of paternal variation in this sample.

Haplogroup J was represented by two subclades, J1-MF260389 and J2-BY114993. J2-BY114993 is phylogenetically close to the ancient individual RISE602 from the Sary-Bel site in the Altai region. This affinity is consistent with the presence of related paternal lineages in Inner Asia during the early Iron Age. The reference phylogeny also includes modern Central Asian individuals within this branch, including a Tajik individual. J1-MF260389 is also represented by an Uzbek individual from Kazakhstan and clusters with the ancient individual H81506 from Jiulongshan, Yuanzhou County, Guyuan City, Ningxia Hui Autonomous Region, China, dated to the Sui–Tang period [23]. These connections indicate broad phylogenetic affinities and do not establish a specific historical migration or continuous local ancestry.

Two individuals were assigned to the G2 subclades G2-FTA54933 and G2-FTA59657. Together with the R1a, R1b, and J lineages, these subclades illustrate the presence of several deeply diverging paternal branches in the NGS dataset.

Haplogroup C2 was represented by four lineages: C2-FT252510, C2-FT411734, C2-ZQ383, and C2-Y148048. Three fall within the broader C2a-F4002 clade, which is widely distributed across Inner Eurasia and among Mongolic-speaking populations [24]. C2-FT252510 is reported in public Y-DNA projects among individuals identified as Kazakh Jalair, whereas C2-FT411734 has been reported among Kazakh Kerey individuals, including members of the Abak subgroup [25]. C2-ZQ383 is closely related to C2a-MF691989, a lineage reported from elite burials associated with the Golden Horde and among Kazakh Tama individuals [26,27]. Both branches descend from ZQ369, a broader clade that also includes ancient individuals from the Tavan Khailast cemetery [28]. C2-Y148048 belongs to a separate C2b branch [29]. These observations document multiple C2 subclades with reported affinities to different Inner Asian groups.

Haplogroup O was represented by three lineages: O1-BY166554 and two O2-M324 subclades, O2-MF9486 and O2-F438. In the reference phylogeny, O2-MF9486 is positioned near individuals from Korea and Japan, whereas O2-F438 belongs to a branch that includes Chinese individuals. These comparisons link the observed O lineages to East Asian paternal variation. The presence of both O1 and O2 also illustrates diversity within haplogroup O in the NGS dataset.

The remaining individuals belonged to N1-FT348418 (*N* = 1) and the Q1 lineages Q1-FT179414 and Q1-YP6182 (one individual each). Haplogroups N and Q have been associated with northern Eurasian, Siberian, and Inner Asian paternal variation. Their occurrence adds further phylogenetic diversity to the NGS dataset.

Together, these high-resolution Y-SNP data reveal substantial subclade diversity among the 19 individuals analyzed. Broad haplogroup categories encompass phylogenetically distinct lineages with different geographic affinities in the available reference data. R1a lineages show connections to western and Inner Eurasian lineages; C2 lineages show connections to several Inner Asian groups; O lineages show East Asian affinities; and J, G2, R1b, N1, and Q1 add further branches of paternal variation.

Given the small size and nonrepresentative sampling of this exploratory dataset (*N* = 19), these observations cannot establish the timing of specific migration events, demonstrate historical continuity, or provide representative estimates of haplogroup frequencies. They illustrate the phylogenetic diversity within broad Y-chromosomal haplogroups and highlight the need for larger, systematically sampled Y-chromosome sequencing studies of Uyghurs from Kazakhstan and neighboring Central Asian populations.

### 2.4. Population Comparison Analysis

Pairwise RST values were calculated for the Kazakhstani Uyghur samples and reference populations represented in YHRD using the 17 Y-STR loci shared across datasets (Supplementary Table S7). Among the four Kazakhstani Uyghur samples, estimates ranged from −0.0047 to 0.0017, with permutation *p*-values ranging from 0.2364 to 0.7267; none of the pairwise comparisons was statistically significant. These near-zero estimates indicate limited paternal genetic differentiation among samples from Almaty, Enbekshikazakh District, Panfilov District, and Uyghur District at the 17 loci examined. Small negative RST estimates can result from sampling variation when the estimated among-population variance component is close to zero. These estimates were retained unchanged in the supplementary matrix and interpreted as providing no evidence of differentiation. Negative estimates were replaced with zero only in the matrix used for ordination.

Comparisons with Uyghur populations from Xinjiang also revealed very low differentiation, with RST estimates ranging from −0.0040 to 0.0085. Only the Enbekshikazakh–Urumqi (RST = 0.0055, *p* = 0.0205) and Panfilov–Korla (RST = 0.0085, *p* = 0.0497) comparisons were nominally significant. Neither remained significant after Bonferroni correction for 300 pairwise tests (α ≈ 0.000167). Thus, no statistically significant differentiation was detected between the sampled Kazakhstani and Xinjiang Uyghur groups after correction for multiple testing.

Pairwise relationships were visualized using two-dimensional Kruskal nonmetric multidimensional scaling (NMDS) of the 17-locus RST matrix (Figure 5). The solution obtained using 100 starting configurations had a normalized Stress-1 value of 0.086. Spearman’s rank correlation between the input dissimilarities and the fitted interpopulation distances was high (ρ = 0.981), consistent with good preservation of their rank order. As a sensitivity analysis, classical multidimensional scaling (principal coordinates analysis; PCoA) was also applied to the same matrix (Supplementary Figure S1). The first two PCoA axes accounted for 88.5% of the sum of positive eigenvalues. Both ordinations showed a similar overall configuration, with Kazakhstani Uyghurs and most Uyghur populations from Xinjiang forming a compact central cluster. The Kazakhstani Uzbek samples were positioned close to this cluster, consistent with the low RST values observed between Kazakhstani Uyghurs and Kazakhstani Uzbeks (RST = 0.0014–0.0246). This proximity is compatible with shared paternal ancestry and historical gene flow among sedentary and oasis-associated populations of southeastern Kazakhstan and adjacent regions.

Comparisons with Kazakh populations revealed greater variation in the degree of differentiation. Among the Kazakh reference populations, the lowest RST values were observed in comparisons with Kazakhs from Southern Kazakhstan (RST = 0.0495–0.0704), Central Kazakhstan (RST = 0.0559–0.0755), East Kazakhstan (RST = 0.0526–0.0826), North Kazakhstan (RST = 0.0833–0.0876), and Xinjiang (RST = 0.0577–0.0897). More pronounced differentiation was observed in comparisons with Kazakhs from West Kazakhstan and Karakalpakstan (RST = 0.1630–0.2498), whereas the highest values were observed for Kazakhs from Altai and Gansu in China (RST = 0.3022–0.3967). The Kizilsu Kyrgyz population also showed substantial differentiation from the Kazakhstani Uyghur samples (RST = 0.1437–0.2222). Of the 40 pairwise comparisons between the four Kazakhstani Uyghur samples and the ten Kazakh and Kyrgyz reference samples, 38 remained statistically significant after Bonferroni correction. In the NMDS ordination, these reference populations occupied peripheral positions relative to the central Uyghur–Uzbek cluster. Kazakhs from Altai, Gansu, West Kazakhstan, and Karakalpakstan, together with Kizilsu Kyrgyz, showed the greatest separation from this cluster. Lineage-specific genetic drift may have contributed to this pattern. Unequal sample sizes and differences in recruitment strategies among the YHRD reference populations may also have affected the magnitude of the estimates.

To assess whether the low differentiation between Kazakhstani Uyghurs and Uyghurs from Xinjiang was also evident at higher marker resolution, we compared Uyghur populations using an expanded panel of 27 Y-STR loci (Supplementary Table S8). RST estimates ranged from −0.0021 to 0.0107 among Kazakhstani Uyghur samples, from −0.0031 to 0.0139 among Uyghur samples from Xinjiang, and from −0.0034 to 0.0150 between the two sets of samples. Four of the 36 pairwise comparisons were nominally significant (*p* = 0.0174–0.0436), but none remained significant after Bonferroni correction (α ≈ 0.001389).

Together, the broader 17-locus comparison and the Uyghur-focused 27-locus analysis indicate low Y-STR differentiation between Kazakhstani Uyghurs and Uyghurs from Xinjiang. These findings are consistent with previous high-resolution Y-STR studies describing the admixed composition of the Uyghur paternal gene pool and its intermediate position between eastern and western Eurasian populations. Within the 17-locus reference panel, Kazakhstani Uyghurs showed the greatest similarity to Xinjiang Uyghurs and Kazakhstani Uzbeks, whereas the Kazakh and Kyrgyz reference populations were more differentiated. The low RST estimates are compatible with shared ancestry, gene flow, or both, but do not by themselves establish historical continuity or identify the timing or direction of demographic events.

### 2.5. Forensic Relevance and Contribution to Reference Datasets

The principal forensic contribution of this study is a geographically documented Y-STR reference dataset for Uyghurs in Kazakhstan. These data are intended to support haplotype-frequency assessment when evaluating male DNA evidence in relevant forensic investigations in Kazakhstan, including comparisons involving male components of mixed samples or paternal-line relationships [2,3]. The high haplotype diversity and low observed haplotype match probability describe the sampled collection; the latter is not a match probability for a particular evidential profile or a case-specific likelihood ratio. Practical interpretation requires an appropriate reference population and consideration of paternal relatedness, sampling uncertainty, and population structure. The present study provides reference data but does not establish their operational adoption by forensic laboratories or validate a casework interpretation procedure.

Buckleton et al. [30] estimated population-specific θ values for forensic match-probability calculations using PowerPlex Y23 haplotypes. The profiles reported here could extend the geographical and population coverage of future surveys by contributing Uyghur reference data from southeastern Kazakhstan. Integration would require harmonization to a common marker set and re-estimation of the relevant statistics, because Yfiler Plus does not include the PowerPlex Y23 markers DYS549 and DYS643. Alternatively, additional typing of these two markers would permit comparison using the complete Y23 panel. No population-specific θ was estimated in the present study, and the pairwise RST estimates reported here should not be substituted for θ in forensic match-probability calculations. The individual profiles provided in Supplementary Table S1 constitute a resource for these future analyses.

## 3. Limitations

Several limitations should be considered when interpreting these findings. First, information on participant ancestry and paternal relationships was obtained through self-reports and oral pedigree interviews. Relatedness was not independently assessed using genome-wide kinship analysis.

Second, the 20-SNP panel provides resolution only at the level of major haplogroups. These categories may encompass multiple terminal subclades, and Y-STR homoplasy can obscure phylogenetic relationships. The median-joining networks therefore provide descriptive summaries of haplotype similarity and should not be interpreted as resolved genealogical trees. They cannot reliably date lineage expansions, identify specific ancestral haplotypes, or determine the timing of historical founder events.

Third, the population comparisons relied on published YHRD reference datasets that varied in sample size and recruitment strategies. These differences, together with the exclusion of atypical Y-STR profiles by the YHRD AMOVA service, may have affected the magnitude of the RST estimates.

Finally, the previously published NGS dataset used for auxiliary phylogenetic comparison comprised only 19 individuals and was insufficient for estimating population-level subhaplogroup frequencies. Future studies would benefit from larger samples collected using clearly documented recruitment strategies and from whole-Y-chromosome sequencing. Forensic application requires an appropriate interpretation framework that accounts for paternal relatedness, reference-sampling uncertainty, and population structure [3,30].

## 4. Materials and Methods

### 4.1. Sample and Data Collection

Peripheral blood samples were collected from unrelated, healthy male Uyghur participants residing in southeastern Kazakhstan during field expeditions in the summer of 2024. The study included four regional groups: Almaty city (*N* = 179), Enbekshikazakh district (*N* = 203), Panfilov district (*N* = 142), and Uyghur district (*N* = 72). Eligibility required self-identification as Uyghur, reported Uyghur ancestry for at least three generations, and no known paternal-line relationship with another participant based on pedigrees extending back at least three generations. These criteria were assessed before enrollment using oral pedigree interviews and written demographic questionnaires. Ancestry was self-reported, and relatedness was not independently assessed using genome-wide kinship analysis. All participants provided written informed consent before sample collection. Blood samples were collected in EDTA-containing tubes and stored until DNA extraction. The study was conducted in accordance with the Declaration of Helsinki (1964) and was approved by the Local Bioethics Committee of the M. Aitkhozhin Institute of Molecular Biology and Biochemistry, Almaty, Kazakhstan, which acted as an external ethics review committee (protocol No. 2, dated 31 October 2023).

For comparative analyses, previously published Uyghur reference samples from Xinjiang, China [12,13,31], were selected using the online comparison service of the Y-Chromosome Haplotype Reference Database (YHRD). These comprised samples from Aksu (*N* = 136; YHRD accession no. YA005703), Karamay (*N* = 119; YA005180), Kashi (*N* = 75; YA005528), Korla (*N* = 131; YA005329), Turpan (*N* = 473; YA005216), and Urumqi (*N* = 439; YA005376), together with a pooled Xinjiang dataset (*N* = 581; YA003847, YA004122, and YA004227). YHRD was used solely as an online comparison service; individual-level records from the reference datasets were neither downloaded nor redistributed.

Additional reference samples were selected from published datasets to place the Uyghur samples in a broader Central Asian context. These included Uzbek populations from Kazakhstan [32], Kazakh populations from different regions of Kazakhstan [33,34,35,36,37], and Kazakh and Kyrgyz populations from China [38,39,40].

### 4.2. DNA Isolation, Amplification, and STR Genotyping

Genomic DNA was extracted from peripheral whole blood using the Wizard^®^ Genomic DNA Purification Kit (Promega, Madison, WI, USA) according to the manufacturer’s protocol. DNA concentration was measured using a Qubit™ 2.0 Fluorometer (Thermo Fisher Scientific, Waltham, MA, USA), and DNA purity was assessed from the A260/A280 absorbance ratio using a NanoDrop™ One spectrophotometer (Thermo Fisher Scientific, USA).

Y-STR profiling was performed using the Yfiler™ Plus PCR Amplification Kit (Thermo Fisher Scientific, USA), which amplifies 27 markers. The multiplex includes the 17-marker Yfiler™ panel (DYS19, DYS385a/b, DYS389I/II, DYS390, DYS391, DYS392, DYS393, DYS437, DYS438, DYS439, DYS448, DYS456, DYS458, DYS635, and Y-GATA-H4) and 10 additional markers: DYS460, DYS481, DYS533, and the rapidly mutating markers DYS449, DYS518, DYS570, DYS576, DYS627, and DYF387S1a/b. DYS385 and DYF387S1 were recorded as unordered allele pairs. The a/b labels indicate ascending allele values in the data table and do not identify the genomic copy carrying each allele.

PCR amplification was performed on a SimpliAmp™ Thermal Cycler (Thermo Fisher Scientific, USA) using the manufacturer’s recommended cycling conditions. Amplified fragments were separated by capillary electrophoresis on an Applied Biosystems 3500 Genetic Analyzer (Thermo Fisher Scientific, USA) using a 36 cm capillary array and POP-4™ polymer (Thermo Fisher Scientific, USA). Fragments were sized against the GeneScan™ 600 LIZ™ Size Standard v2.0 (Thermo Fisher Scientific, USA).

Alleles were called using GeneMapper™ ID-X software, version 1.6. Each amplification batch included positive (Control DNA 007) and negative (ddH_2_O) controls. All atypical allele calls, including microvariants and putative null alleles, were confirmed by a second PCR amplification of the original DNA extract and manual review of the electropherograms. These profiles were retained in the descriptive dataset, and their inclusion in population comparisons was governed by the YHRD AMOVA service’s validation rules.

Laboratory quality control included participation in the YHRD quality control program. In accordance with recommendations for reporting Y-STR population data [41], all haplotypes generated in this study were submitted to YHRD (https://www.yhrd.org/, accessed on 15 August 2026) under accession numbers YA006051, YA006052, YA006053, and YA006054. The complete haplotype dataset is provided in Supplementary Table S1.

Major Y-chromosomal haplogroups were assigned by genotyping 20 Y-SNP markers (F1067, F1756, F1918, L901, M119, M122, M124, M170, M172, M174, M184, M20, M201, M231, M242, M267, M343, M420, M48, and M96) using TaqMan probes on a QuantStudio 5 instrument (Thermo Fisher Scientific, USA), according to the manufacturer’s protocol. Reactions with ambiguous amplification curves or signals below the detection threshold were repeated using the original DNA extract. After repeat testing, unambiguous major haplogroup assignments were obtained for all 596 individuals, yielding a final haplogroup assignment rate of 100%. Haplogroup nomenclature followed the ISOGG Y-DNA Haplogroup Tree (https://isogg.org/tree/ (accessed on 1 April 2026)).

### 4.3. Data Analysis

Electropherograms were processed using GeneMapper™ ID-X software, version 1.6 (Thermo Fisher Scientific, USA). Alleles were called against the manufacturer’s allelic ladders and manually reviewed. The resulting profiles were assembled into multilocus Y-STR haplotypes for population genetic analyses.

Haplotype frequencies were estimated by direct counting using Arlequin v3.5 [42]. Summary statistics included the number of distinct haplotypes, the proportion of individuals with a haplotype observed only once, haplotype diversity (HD), haplotype match probability (HMP), and discrimination capacity (DC). Haplotype diversity was calculated using the unbiased estimator HD = (n(1 − ∑p_i^2^))/(n − 1), where n is the sample size and pi is the frequency of the ith haplotype [43]. The observed HMP was calculated as ∑p_i^2^, and DC was calculated as the number of distinct haplotypes divided by the sample size. Locus-specific gene diversity (GD) and polymorphism information content (PIC) were calculated using STRAF v2.2.2 [44]. Single-locus random match probabilities, power of exclusion values, and typical paternity indices were not used to interpret the forensic evidential value of Y-chromosomal profiles.

Population differentiation was quantified using pairwise RST estimates obtained through the YHRD analysis of molecular variance (AMOVA) service [45]. RST incorporates differences in STR repeat numbers within a stepwise mutation framework. Molecular distances were based on squared differences in repeat numbers, and AMOVA partitioned this variation into within-population and among-population components. RST expresses the relative contribution of the among-population component to the total variation.

Two marker panels were analysed separately. The broader comparison used the 17-marker Yfiler panel: DYS19, DYS385a/b, DYS389I, DYS389II, DYS390, DYS391, DYS392, DYS393, DYS437, DYS438, DYS439, DYS448, DYS456, DYS458, DYS635, and Y-GATA-H4 (Supplementary Table S7). The Uyghur-focused comparison used the full 27-marker Yfiler Plus panel, which additionally includes DYS449, DYS460, DYS481, DYS518, DYS533, DYS570, DYS576, DYS627, and DYF387S1a/b (Supplementary Table S8). The 17-marker and 27-marker matrices comprised 25 and nine population samples, respectively.

The YHRD implementation corrected DYS389II repeat counts by subtracting DYS389I to avoid counting variation in the shared repeat segment twice. DYS385a/b and, for the 27-marker analysis, DYF387S1a/b were handled as unordered allele pairs without assigning alleles to specific genomic copies. The service excluded profiles with missing or unspecified alleles, nonstandard allele designations, intermediate alleles, null alleles, duplications outside DYS385 and DYF387S1, or three or more allele copies at a locus.

The null hypothesis of no differentiation between each pair of population samples was assessed using 10,000 permutations. Bonferroni correction was applied separately to all pairwise tests within each matrix. The 25-sample comparison involved 300 tests, giving a significance threshold of 0.05/300 = 0.0001667; the nine-sample comparison involved 36 tests, giving a threshold of 0.05/36 = 0.0013889. Unadjusted permutation *p*-values and the original RST estimates, including negative estimates, are reported in Supplementary Tables S7 and S8. Small negative estimates were interpreted as sampling variation around zero differentiation and were replaced with zero only when preparing the dissimilarity matrix for ordination.

Pairwise relationships were visualized using two-dimensional Kruskal nonmetric multidimensional scaling (NMDS) [46]. The analysis used 100 starting configurations. Model fit was assessed using normalized Stress-1 and Spearman’s rank correlation between the input dissimilarities and the fitted interpopulation distances. As a sensitivity analysis, classical multidimensional scaling (principal coordinates analysis; PCoA) was applied to the same matrix, and the ordination is presented in Supplementary Figure S1.

Haplotype relationships within R1a and J2 were visualized using median-joining networks constructed in NETWORK v5.0.1.0 and displayed using NETWORK Publisher [47]. Analyses used a subset of 15 single-copy Y-STR markers. DYS389II values were corrected by subtracting DYS389I repeat counts to avoid counting variation in DYS389I twice. The multicopy loci DYS385a/b and DYF387S1a/b were excluded because alleles could not be assigned to specific genomic copies. DYS449, DYS460, DYS481, DYS518, DYS533, DYS570, DYS576, and DYS627 were also omitted to limit network reticulation. Networks were constructed with ε = 0 and a weight of 10 for each locus. Within each haplogroup, network reconstruction was based exclusively on STR haplotypes, without additional SNP-based phylogenetic constraints.

STR-based haplogroup predictions were generated using the NevGen Y-DNA haplogroup predictor, which applies a Bayesian framework based on reference datasets (https://www.nevgen.org/; accessed on 1 April 2026). These predictions were used only as an auxiliary check. Major haplogroup frequencies reported in the main analyses were based on TaqMan SNP genotyping.

Previously published NGS-based Y-SNP data from 19 Kazakhstani Uyghur males [19] were reassessed using FamilyTreeDNA Discover™ (https://discover.familytreedna.com/; accessed on 1 April 2026) to refine the terminal or subterminal placement of paternal lineages in the Y-chromosome phylogeny. These data were used only for auxiliary phylogenetic comparisons with available modern and ancient lineages and were not used to estimate population-level haplogroup frequencies or support other formal population-level inferences.

## 5. Conclusions

This study presents the first large-scale paternal genetic characterization of 596 Uyghur males from southeastern Kazakhstan using a panel of 27 Y-STRs and 20 core Y-SNPs. The analyzed population exhibits exceptionally high paternal genetic diversity (HD = 0.9993) and a highly heterogeneous haplogroup composition. The coexistence of predominant western and Inner Eurasian lineages (such as R1a, J2, and R1b) with eastern and northern Eurasian components (such as C2, O2, and Q) underscores a complex paternal genetic architecture rather than descent from a single dominant founder.

Median-joining network analyses of the predominant haplogroups, supported by R_ST_-based distance metrics, indicate an absence of pronounced regional subdivision among the four sampled Kazakhstani locations, pointing to a shared paternal background and extensive internal gene flow. Crucially, population comparisons utilizing both 17- and 27-locus Y-STR matrices revealed no statistically significant differentiation between Kazakhstani Uyghurs and Uyghur populations from Xinjiang after correction for multiple testing. In the broader Central Asian genetic landscape, these Uyghur groups form a cohesive cluster alongside neighboring sedentary populations, remaining clearly distinct from strongly clan-structured nomadic groups. Furthermore, exploratory high-resolution Y-SNP analysis confirmed that this macroscopic diversity is underpinned by multiple deeply diverged terminal lineages.

Collectively, these findings provide a robust, highly discriminatory reference dataset for forensic genetic applications in a previously underrepresented region. Beyond forensic utility, the data establish a comprehensive descriptive baseline demonstrating a continuous paternal genetic landscape across the Kazakhstan–Xinjiang corridor. Future studies incorporating explicitly structured sampling and whole-Y-chromosome sequencing will be essential to further resolve the fine-scale terminal phylogeny and temporal dynamics of these Central Eurasian lineages.

## Figures and Tables

**Figure 1:**
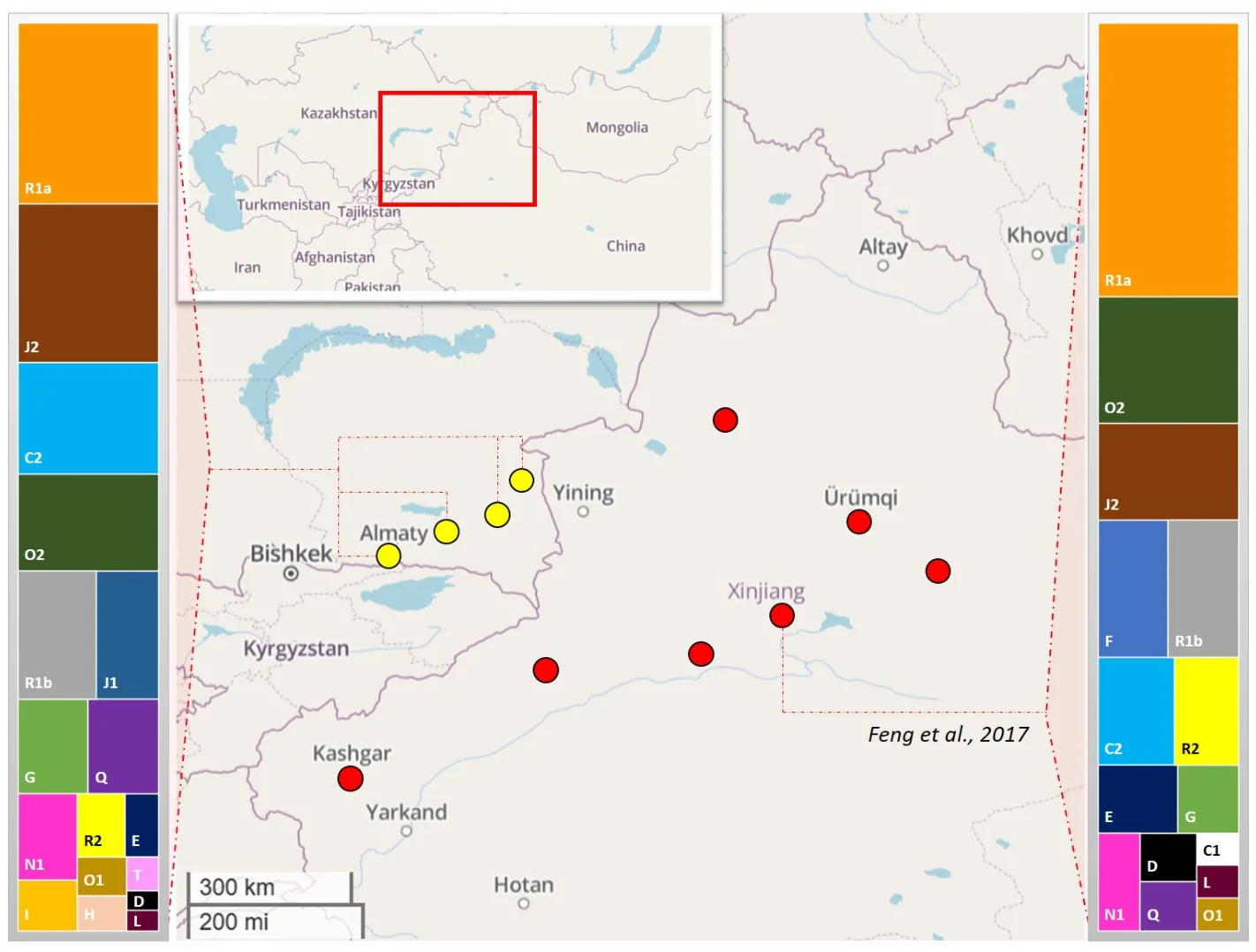
Sampling locations and major Y-chromosomal haplogroup frequencies in the Uyghur populations analyzed in this study. Yellow circles indicate the locations of newly collected Uyghur samples in southeastern Kazakhstan; red circles indicate previously published Uyghur samples from Xinjiang, China [10] included in the comparative population genetic analyses using YHRD data. Stacked bar plots show the relative frequencies of major Y-chromosomal core haplogroups in the corresponding populations. The inset map shows the location of the study area within Central Asia. The base map adapted from Open Street Map. The underlying map data are available under the Open Database License.

**Figure 2:**
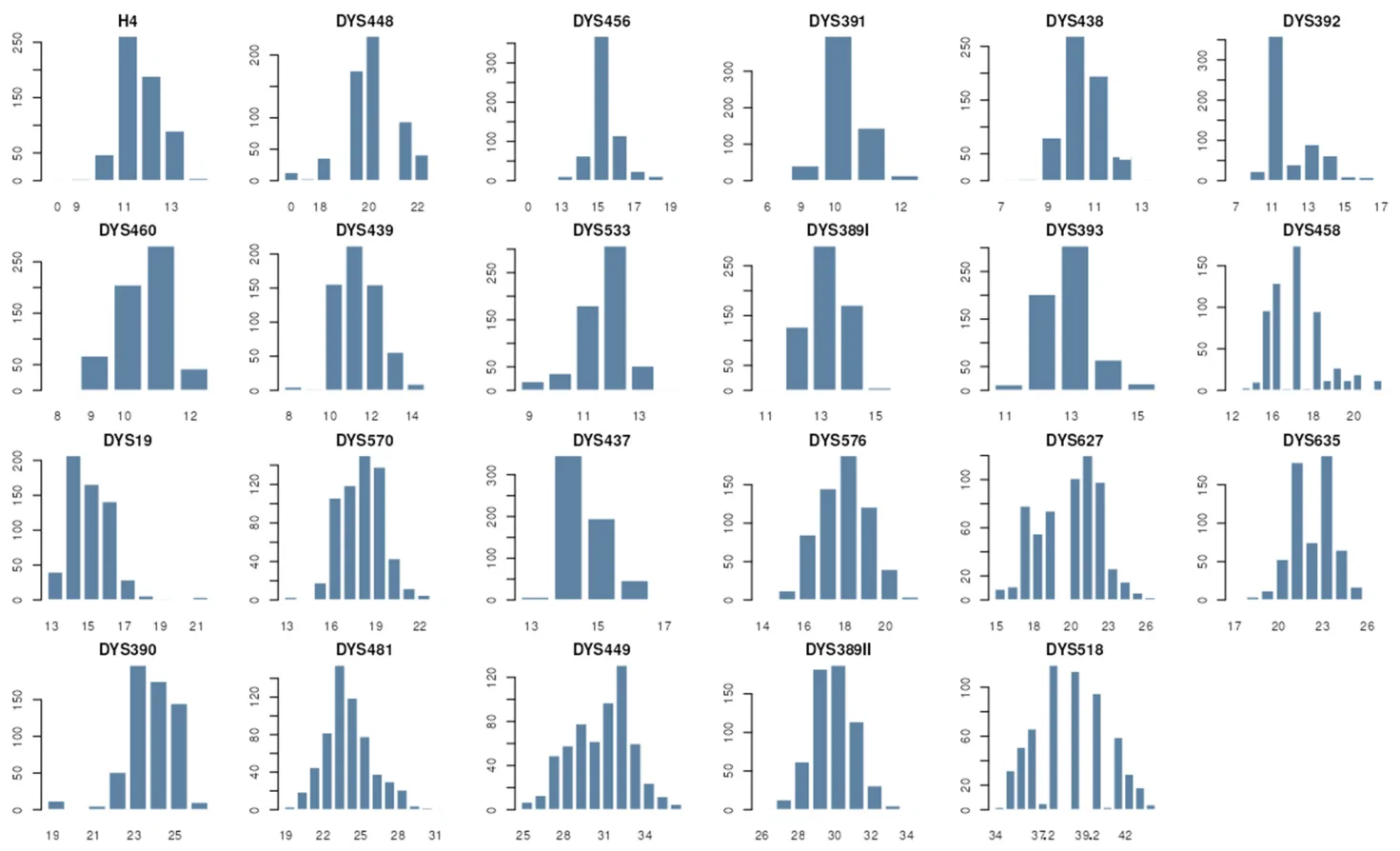
Allele frequency distributions at 23 single-copy Y-STR loci in the Uyghur sample from Kazakhstan. The horizontal axes show allele designations, and the vertical axes show allele frequencies.

**Figure 3:**
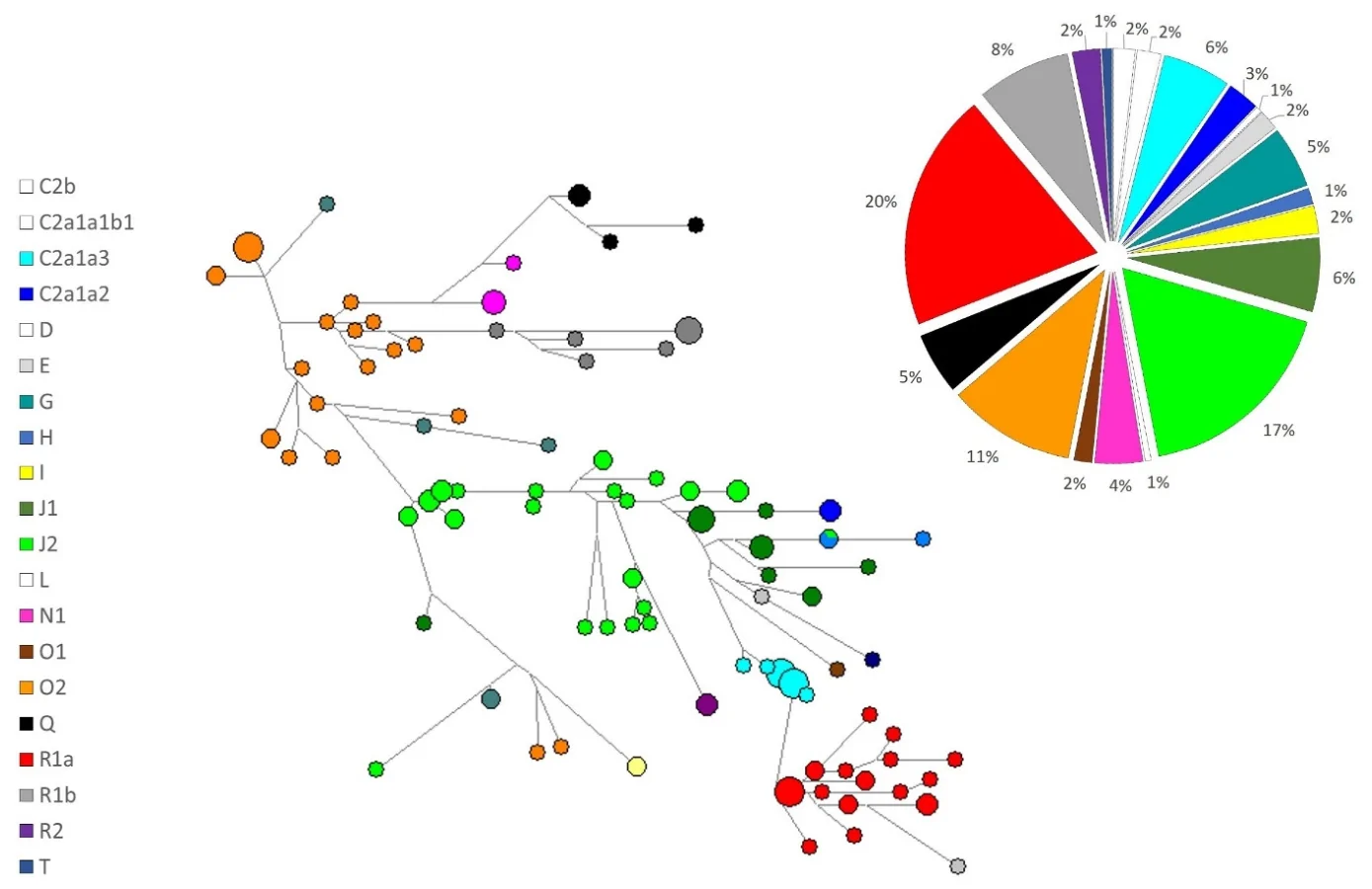
Median-joining network of 15-locusY-STR haplotypes in the Uyghur sample from Kazakhstan. Node sizes are proportional to haplotype frequencies, and branch lengths represent mutational steps. Colors indicate Y-chromosomal haplogroups (see legend). Singleton haplotypes are omitted from the network visualization; consequently, some low-frequency haplogroups are not shown in the network. These haplogroups are represented by white segments in the haplogroup frequency diagram.

**Figure 4:**
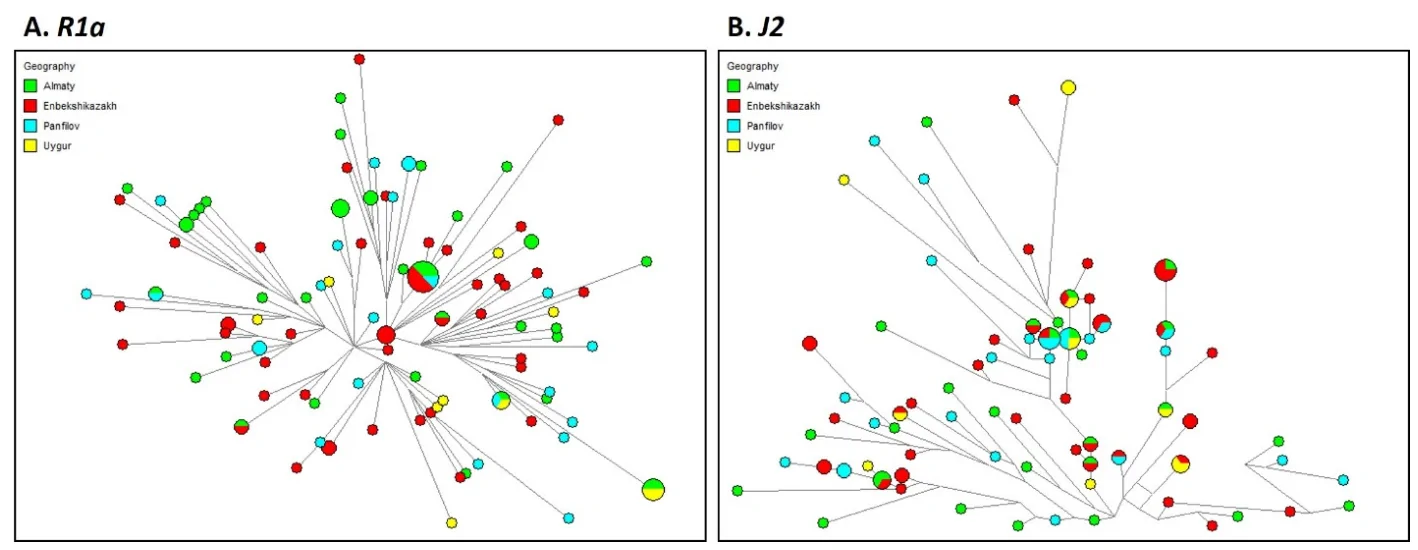
Median-joining networks of Y-STR haplotypes within the two most frequent haplogroups in the Uyghur sample from Kazakhstan: (**A**) R1a and (**B**) J2. The networks were constructed using 15 Y-STR loci, excluding DYS385a/b. Each circle represents a haplotype, and its size is proportional to haplotype frequency. Lines connecting nodes represent mutational steps. Colors indicate the sampling location: Almaty city or Enbekshikazakh, Panfilov, or Uyghur District. All observed haplotypes were included in the network reconstruction.

**Figure 5:**
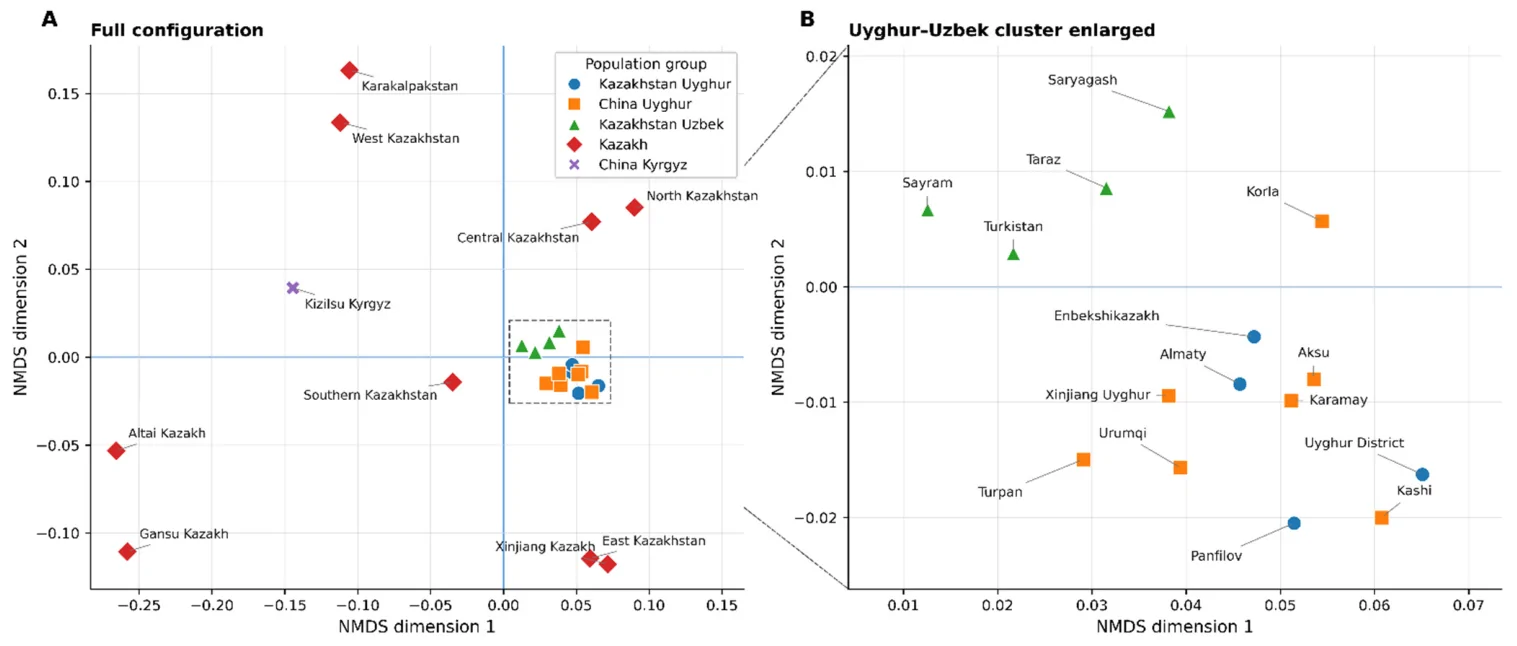
Two-dimensional nonmetric multidimensional scaling (NMDS) of pairwise RST estimates among 25 populations based on 17 Y-STR loci. **Panel A** shows the full ordination, with labels displayed for populations outside the central cluster. The dashed rectangle outlines the region enlarged in **Panel B**. Symbols and colors denote population groups. Negative RST estimates were set to zero for ordination; the original estimates are reported in Supplementary Table S7.

**Table 1:** Haplotype diversity and forensic parameters based on 27 Y-STR markers in Uyghur samples from Kazakhstan.

Population	Number of Samples	Number of Distinct Haplotypes	Frequency of Unique Haplotypes	Discrimination Capacity	Haplotype Match Probability	Haplotype Diversity
Almaty	179	170	0.9050	0.9497	0.0062	0.9994
Enbekshikazakh	203	187	0.8522	0.9212	0.0058	0.9991
Panfilov	142	132	0.8803	0.9296	0.0084	0.9986
Uygur	72	62	0.7639	0.8611	0.0193	0.9945
Total Kazakhstan	596	515	0.7617	0.8641	0.0023	0.9993

## Data Availability

The individual Y-STR haplotypes generated in this study are provided in Supplementary Table S1. Pairwise RST estimates and corresponding permutation *p*-values are reported in Supplementary Tables S7 and S8. The study haplotypes were submitted to the Y-Chromosome Haplotype Reference Database (YHRD) under accession numbers YA006051, YA006052, YA006053, and YA006054. Comparative YHRD reference samples were accessed through the online analysis service.

## Supplementary Materials

The following supporting information can be downloaded at: https://www.mdpi.com/article/10.3390/ijms27198641/s1.
